# Cytoprotective effects of Avenathramide C against oxidative and inflammatory stress in normal human dermal fibroblasts

**DOI:** 10.1038/s41598-019-39244-9

**Published:** 2019-02-27

**Authors:** Chenxuan Wang, Christopher H. Eskiw

**Affiliations:** 1 Department of Food and Bioproduct Sciences, University of Saskatchewan, Saskatoon, Canada; 2 Department of Biochemistry, Microbiology and Immunology, University of Saskatchewan, Saskatoon, Canada

## Abstract

Natural polyphenols are promising anti-aging compounds not only for their antioxidant activity, but also their ability to activate specific cellular pathways mediating the aging process. Avenanthramide C (Avn C), found exclusively in oats, is a natural antioxidant associated with free radical scavenging; however, it is how this compound elicits other protective effects. We investigated the intracellular antioxidant activity of Avn C and other cytoprotective potential in normal human skin fibroblasts exposed to extracellular stress. Avn C reduced H_2_O_2_-induced oxidative stress by reducing intracellular free radical levels and antioxidant gene transcripts. Avn C also resulted in decreased levels of gene transcripts encoding pro-inflammatory cytokines in response to H_2_O_2_ or tumor necrosis factor-α (TNF-α). This reduction in cytokine gene transcription occurred concomitantly with reduced phosphorylated nuclear factor-κB (NF-κB) p65, and decreased NF-κB DNA binding. Avn C further induced *heme oxygense-1 (HO-1)* expression through increased Nrf2 DNA binding activity, demonstrating a second mechanism by which Avn C attenuates cellular stress. Collectively, our findings indicate that Avn C protects normal human skin fibroblasts against oxidative stress and inflammatory response through NF-κB inhibition and Nrf2/HO-1 activation.

## Introduction

**Figure 1:**
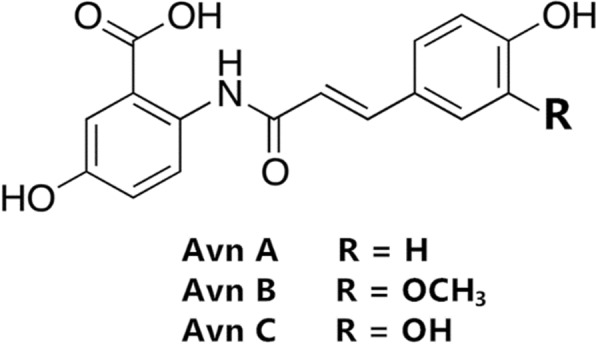
Chemical structures of Avn A, B and C.

Oats (*Avena sativa* L.) have many scientifically verified health-promoting effects, making them one of the best grains for human consumption^1,2^. Majority of the health benefits of oats are attributed to the cholesterol-lowering potential of β-glucan^3^; however, several phytochemicals have also been found and recognized for their positive impacts on human health, which include phenolic compounds^4^. Since most phenolics are found in the bran layer of oat grains, oats could be a significant source of dietary antioxidants as they are normally consumed as whole-grain cereal^4,5^. Major compounds that exhibit antioxidant activity in oats are vitamin E (tocols), phytic acid, phenolic acids, and avenanthramides (Avns)^4,6^. Avns have been receiving increased attention in recent years because they are a group of unique phenolic compounds found exclusively in oats and exhibit high antioxidant activity^7^. Avns are conjugates of a phenylpropanoid (p-coumaric, ferulic, or caffeic acid) with anthranilic acid or 5-hydroxy anthranilic acid^7^. The majority of Avns can be found in oat groats, with the highest concentration in the bran. More than 20 different forms; however three specific Avns; Avn A, Avn B and Avn C^8^ (Fig. 1) Avns have been identified and make up the vast majority of this.

Avns were firstly discovered in 1989^7^. Subsequently, Dimberg *et al*.^9^ measured their antioxidant activity by the inhibition of oxygen consumption in a linoleic acid system and reported that their activity was 10–30 times higher than those of caffeic acid, ferulic acid and vanillin isolated from oats. Since then, a number of studies have been focused on investigating health promoting effect of Avn or Avn-enriched oat extracts (see^10^ for a comprehensive review). It has been found that Avns showed strong antioxidant both *in vitro* and *in vivo* with Avn C being the highest^4,9–14^. Moreover, several studies have indicated that both oat phenolic-rich extract and pure Avns had anti-inflammatory activity by reducing the expression of pro-inflammatory cytokines and repressing NF-κB function^15–18^. These studies document the anti-inflammatory response in cancer cell lines and; therefore, it remains unclear if Avn C is able to supress pro-inflammatory signals in normal cells/tissues. In addition, Avn C was also reported to inhibit the proliferation of rat vascular smooth muscle cells through modulation of cell cycle, indicating the potential health benefit of oat consumption in the prevention of coronary heart disease^19,20^. Notably, the structures of Avns are very similar to a synthetic anti-allergic drug called Tranilast, which also has been found to have anti-proliferative effect on rat vascular smooth muscle cells^21^. Recently, the anti-proliferative effects of Avns have also been examined on several cancerous cell lines and found that Avn C was able to decrease the proliferative rates of colonic cancer cell lines, including Caco-2, HT29, LS174T, HCT116^22^ and MDA-MB-231 breast cancer^23^.

Most natural antioxidants found in our diet exhibit few side effects in humans due to their rapid metabolic rate^24,25^. Several studies have shown that Avn C had strong antioxidant property through its free radical scavenging *in vitro*^4,9,13,18^; however, is this the only potential role for Avn C inside cells? Previous cellular studies have largely focused on immortalized or cancer cell lines^8^ to demonstrate the potential use of these compounds in killing or inhibiting the growth of cells with aberrant function. Animal studies have focused on the therapeutic potential of Avns in disease^15–17^. Interestingly, Avn C has also been demonstrated to activate the nuclear factor erythroid 2-krupple-like 2 (Nrf-2) signalling in immortalized kidney cells^26^; a factor associated with pro-longevity. These studies have indicated anti-oxidant, anti-inflammatory, anti-genotoxic and anti-proliferation mechanisms/cellular pathways, which could cooperate and lead to overall increases in health and lifespan^27,28^. Detailed information into how Avn C impact specific cellular pathways in normal healthy tissues may provide insight into how this compound may promote increased cellular health.

To investigate cytoprotective effect of Avn C on normal dermal fibroblasts, a range of Avn C concentrations were tested in 2DD cells followed by exposure to extracellular stress. We found that Avn C penetrated cells and reduce the levels of intracellular free radicals induced by H_2_O_2_, and reduce transcripts encoding H_2_O_2_-induced antioxidant enzymes and pro-inflammatory cytokines. Avn C pre-treatment also attenuated the production pro-inflammatory marker transcripts in the presence of TNF-α stimulation. The reduction of pro-inflammatory transcripts occurred concomitantly with reduced phosphorylated nuclear factor-κB (NF-κB) p65. In addition, Avn C induced *HO-1* expression via Nrf2 DNA binding activity and reduced pro-inflammatory markers via decreased NF-κB-DNA binding. Additionally, Avn C decreased the proliferative rates of 2DD fibroblasts without causing cell death through autophagy-independent pathway as Avn C did not increase the amount of the autophagy marker protein light chain 3 isoform II (LC3-II), phosphorylated mTOR and SIRT1. Taken together, we demonstrate that Avn C attenuated the effects of H_2_O_2_ and TNF-α through Nrf2/HO-1 activation and NF-κB inhibition as well as anti-proliferative effect in human skin fibroblasts.

## Results

### **Avn C protects normal human skin fibroblasts against H**_**2**_**O**_**2**_-induced oxidative stress and DNA damage

**Figure 2:**
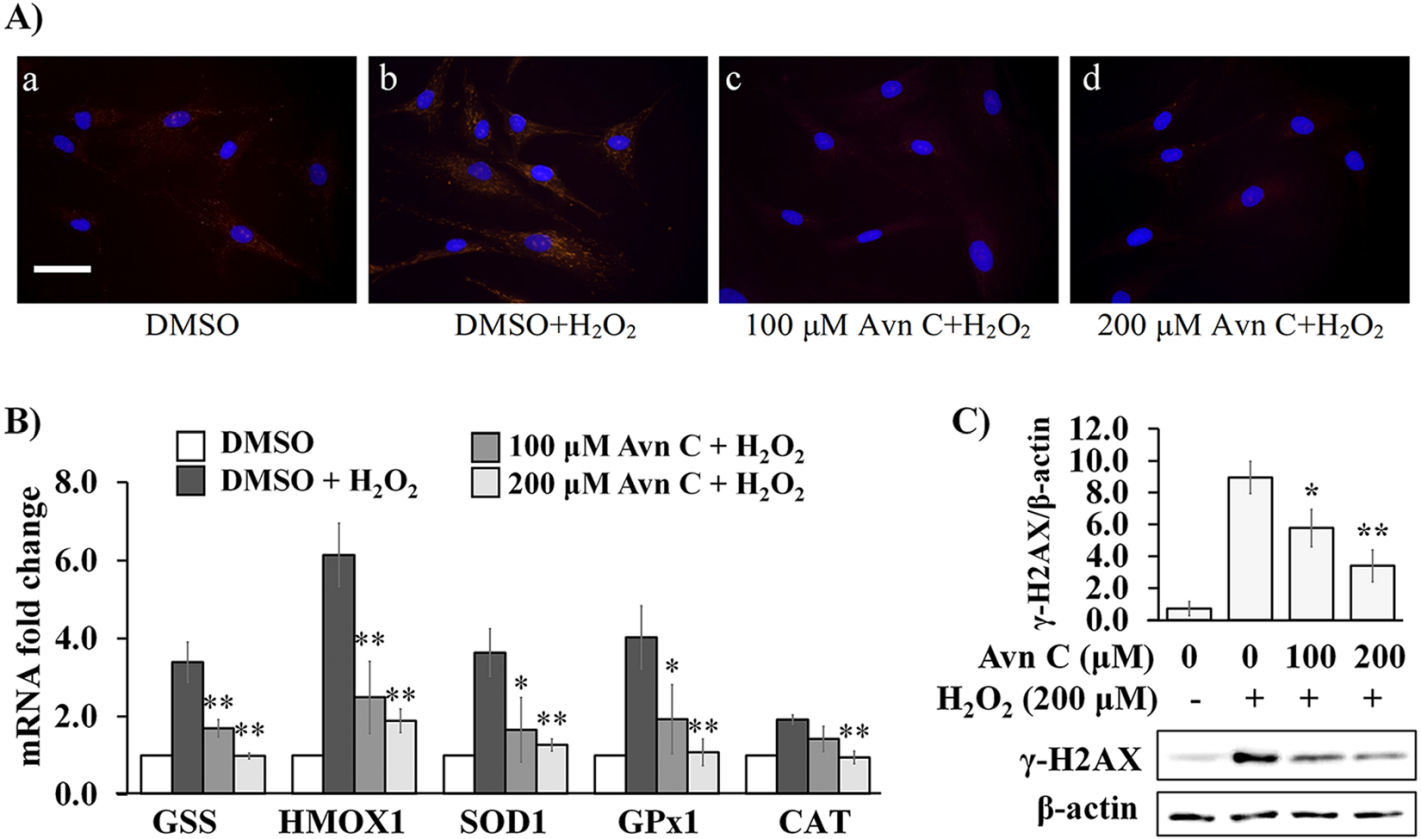
Avn C reduces H_2_O_2_-induced oxidative stress and DNA double-strand damage in 2DD fibroblasts. Normal human skin fibroblasts (2DD) were pre-treated with DMSO (0) or Avn C (100 μM and 200 μM) for 48 h followed by 1 h exposure to 200 μM H_2_O_2_. (**A**) Representative images from cells stained with MitoTracker Orange dye. MitoTracker Orange reacts with free radicals inside cells to generate orange fluorescence. Cell chromatin is counterstained with DAPI (blue) to indicate nuclear location. Scale bar: 50 μm. (**B**) qRT-PCR on cDNA libraries was performed for *glutathione synthetase (GSS), heme oxygenase 1 (HMOX1), superoxide dismutase 1 (SOD1), glutathione superoxidase 1 (GPx1)* and *catalase (CAT)* gene transcripts. Data are represented as the fold change against control. (**C**) Western blot analysis of γ-H2AX expression. Data are presented as a ratio of γ-H2AX to β-actin. Representative blot images are shown. All graphs indicate mean values from three biological replicates. Error bars represent standard deviations. *p value < 0.05, **p value < 0.01 vs H_2_O_2_ treatment alone.

Avn C has been shown to process strong antioxidant activity by *in vitro* extracellular assays^4,9,13,18^; however, it is unclear how well this phenolic compound penetrates and is able to scavenge free radicals in normal human cells. To evaluate cytoprotective effect of Avn C against H_2_O_2_-induced oxidative stress, normal human fibroblasts (2DD) cells were pre-treated with Avn C for 48 hours prior to 1 h exposure to H_2_O_2_. H_2_O_2_ treatment is documented to induce free radicals in cell culture experiments at wide range of concentrations and times^28–30^. We selected 200 μM for 1 hour to induce a strong response but not so acute as to be prevent cells from recovery. To determine the levels of free radicals, fixed cells were incubated with a reduced, non-fluorescent dye MitoTracker Orange. When this dye interactions with free radicals, it is oxidized and becomes fluorescence. Fluorescence micrographs were collected and the intensity of the Mitotracker dye was examined (Fig. 2A). Control cells (DMSO only) showed a small amount of orange fluorescent signal (Fig. 2Aa) and a dramatic increase of the signal was observed in the cells with H_2_O_2_ treatment alone (Fig. 2Ab). Pre-treatment with 100 and 200 μM Avn C for 48 h following H_2_O_2_ treatment showed decreased orange fluorescent signals (Fig. 2Ac,d) compared to H_2_O_2_ treatment alone, indicating Avn C was capable of penetrating into cells and scavenging intracellular free radicals.

To further evaluate the protective capacity of Avn C against oxidative stress in human fibroblast cells, we examined transcript levels of anti-oxidant genes normally stimulated by oxidative stress. Anti-oxidant enzymes are induced in response to H_2_O_2_ treatment, including *glutathione synthetase (GSS)*^18^, *heme oxygenase 1 (HMOX1)*^31^, *superoxide dismutase 1 (SOD1)*^32^, *glutathione peroxidase 1 (GPx1)*^33^ and *catalase (CAT)*^34^. After pre-treatment with Avn C for 48 h followed by 1 h H_2_O_2_ exposure, total RNA was extracted, converted to cDNA and evaluated by qRT-PCR. H_2_O_2_ treatment without pre-treatment of Avn C resulted in a significant increase in the transcripts from the *GSS, HMOX1, SOD1, GPx1* and *CAT* gene expression (Fig. 2B). By comparing cells with H_2_O_2_ treatment alone, pre-treatment of 2DD cells with 200 μM Avn C significantly decreased all the transcripts induced by H_2_O_2_. Furthermore, pre-treatment with 100 μM Avn C significantly decreased the levels of all the transcripts with an exception of *CAT* compared to H_2_O_2_ treatment alone. Additionally, one of the major consequences of free radical production and exposure is the increased DNA damage. We evaluated the ability of Avn C to protect cells from H_2_O_2_-induced free radical damage. Upon DNA damage the histone variant γ-H2AX becomes phosphorylated and deposited at the sites of damage, facilitating the recruitment of repair machinery^35^. We observed that pre-treatment with Avn C (100 μM and 200 μM) decreased levels of γ-H2AX by western blotting (Fig. 2C), further demonstrating the cytoprotective effects of Avn C in primary dermal fibroblasts. These observations indicate that Avn C is able to cross into cells and scavenge free radicals resulting in a decreased in anti-oxidant transcript levels as well as decreased γ-H2Ax protein.

### Avn C protects normal human skin fibroblasts against H_2_O_2_-induced pro-inflammatory signalling

**Figure 3:**
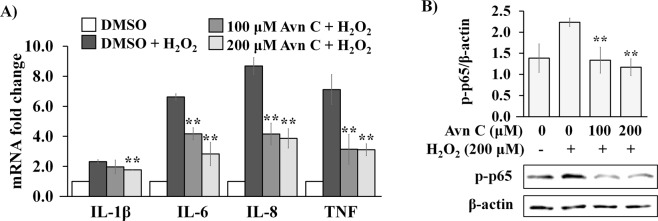
Avn C suppresses H_2_O_2_-induced mRNA expression of pro-inflammatory cytokines and NF-κB activation in 2DD fibroblasts. Normal human skin fibroblasts (2DD) were pre-treated with DMSO (0) or Avn C (100 μM and 200 μM) for 48 h followed by 1 h exposure to 200 μM H_2_O_2_. (**A**) qRT-PCR on cDNA libraries was performed for *interlukin-1 beta* (*IL-1β*), *interlukin-6* (*IL-6*), *interlukin-8* (*IL-8*) and *tumor necrosis factor (TNF)* gene transcripts. Data are represented as the fold change against control. (**B**) Western blot analysis of phospho-NF-*κ*B p65 (Ser536) (p-p65) expression. Data are presented as a ratio of p-p65 to β-actin. Representative blot images are shown. All graphs indicate mean values from three biological replicates. Error bars represent standard deviations. *p values < 0.05, **p values < 0.01 vs H_2_O_2_ treatment alone.

Increased levels of free radicals and oxidative stress inside cells are strongly associated with increased inflammatory response^36,37^. As we observed that Avn C showed cytoprotective effect against H_2_O_2_-induced oxidative stress in 2DD normal human skin fibroblasts, we evaluated if Avn C can also reduce H_2_O_2_-induced pro-inflammatory signaling in 2DD cells. We first measured the ability of Avn C to suppress pro-inflammatory cytokines following H_2_O_2_ treatment. Transcripts from *Il-1β, Il-6, Il-8* and *TNF-α* genes were evaluated by qRT-PCR. Avn C significantly reduced the amounts of transcripts from *Il-6, Il-8* and *TNF-α* under pre-treatment conditions of 100 μM and 200 μM Avn C, whereas a significant reduction of *Il-1β* was only observed at 200 μM pre-treatment concentrations (Fig. 3A).

Oxidative stress is also strongly associated with pro-inflammatory signalling. Free radicals cause cells to produce inflammatory markers through direct or indirect way to activate NF-*κ*B, a dimer of p65 (RelA) and p50 proteins, driving the expression of pro-inflammatory cytokines^38,39^. As we observed that Avn C suppressed H_2_O_2_-induced pro-inflammatory cytokine transcripts, we further evaluated if this suppression effect was mediated by inhibition of NF-κB activation. Western blot analyses of phosphorylated NF-*κ*B p65 (Ser536) demonstrated that H_2_O_2_ treatment alone resulted in an increased protein level of phosphorylated p65, which was significantly reduced by 100 μM and 200 μM Avn C pre-treatment (Fig. 3B). These observations indicate that Avn C is able to protect normal human skin fibroblasts against H_2_O_2_-induced inflammation through suppression of increased levels of pro-inflammatory cytokine transcription and NF-*κ*B activation.

### Avn C attenuates TNF-α-induced responses normal human skin fibroblasts

**Figure 4:**
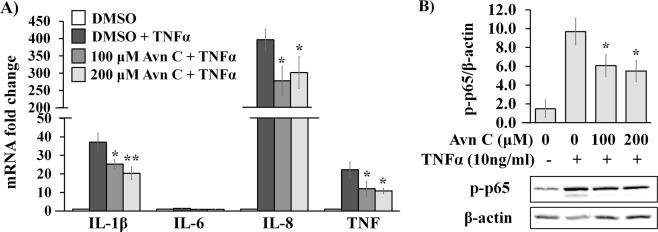
Avn C suppresses TNF-α-induced mRNA expression of pro-inflammatory cytokines and NF-κB activation in 2DD fibroblasts. Normal human skin fibroblasts (2DD) were pre-treated with DMSO (0) or Avn C (100 μM and 200 μM) for 24 h followed by 4 hours exposure to 10 ng/mL TNF-α. (**A**) qRT-PCR on cDNA libraries was performed for *interlukin-1 beta* (*IL-1β*), *interlukin-6* (*IL-6*), *interlukin-8* (*IL-8*) and *tumor necrosis factor alpha (TNF)* gene transcripts. Data are represented as the fold change against control. (**B**) Western blot analysis of phospho-NF-*κ*B p65 (Ser536) (p-p65) expression. Data are presented as a ratio of p-p65 to β-actin. Representative blot images are shown. All graphs indicate mean values from three biological replicates. Error bars represent standard deviations. *p values < 0.05, **p values < 0.01 vs TNF-α treatment alone.

As we observed the protective effect of Avn C against oxidative stress-induced inflammatory response from exogenous free radicals in 2DD fibroblasts, we investigated if the Avn C could also suppress pro-inflammatory signalling induced by TNF-α. TNF-α can function as either an autocrine or paracrine stimulator that binds receptor and triggers signaling, leading to NF-*κ*B activation in different cell types^40^. Hence, we evaluated Avn C ability to protect 2DD fibroblasts against TNF-α-induced production of pro-inflammatory markers. qRT-PCR results demonstrate that TNF-α treatment alone strongly increased the level of transcripts from the *Il-1β, Il-8* and *TNF-α* genes (Fig. 4A). Pre-treatment with Avn C at 100 μM and 200 μM significantly reduced the mRNA levels *Il-1β*, *Il-8* and *TNF-α* by TNF-α treatment. Western blot analyses of phosphorylated NF-*κ*B p65 (Ser536) demonstrated that TNF-α treatment alone resulted in an increased level of phosphorylated p65, which was significantly reduced by 100 μM and 200 μM Avn C pre-treatment (Fig. 4B). These observations indicate that Avn C attenuates TNF-α-mediated increases in inflammatory markers 2DD fibroblasts.

### Avn C reduces pro-inflammatory cytokine transcripts through reduced DNA binding activity of NF-κB in normal human skin fibroblasts

**Figure 5:**
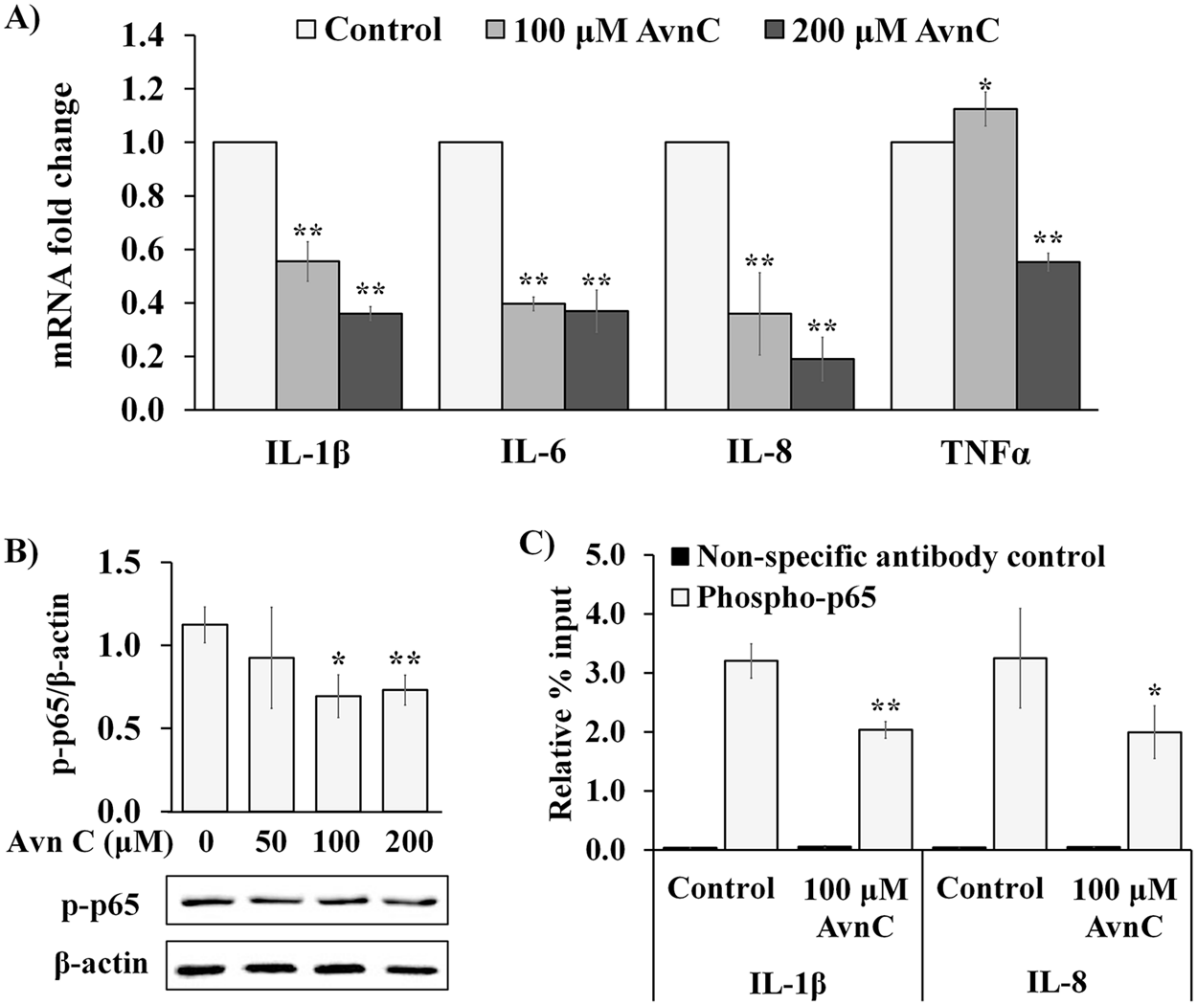
Avn C reduces pro-inflammatory cytokine transcriptions through decreased DNA binding activity of NF-κB in 2DD fibroblasts. (**A**) 2DD human skin fibroblasts were treated with DMSO (control) or Avn C (100 μM and 200 μM) for 48 h. qRT-PCR on cDNA libraries was performed for *interlukin-1 beta* (*IL-1β*), *interlukin-6* (*IL-6*), *interlukin-8* (*IL-8*) and *tumor necrosis factor (TNF)* gene transcripts. Data are represented as the fold change against control. (**B**) Western blot analysis of phospho-NF-κB p65 (p-p65) expression in 2DD cells treated with DMSO (0) or Avn C (50 μM, 100 μM and 200 μM) for 48 h. Data are presented as a ratio of p-p65 to β-actin. Representative blot images are shown. (**C**) ChIP-qPCR analysis of phospho-NF-κB p65 binding activity to promoter regions of *interlukin-1 beta* (*IL-1β*) and *interlukin-8* (*IL-8*) in 2DD cells treated with control (DMSO) or 100 μM Avn C for 48 h. Data are represented as the enrichment relative to % input. All graphs indicate mean values from three biological replicates. Error bars represent standard deviations. *p values < 0.05, **p values < 0.01 vs control.

Constitutive low-grade inflammation is tightly associated with ageing process, most of which focused on the activation of NF-κB system in various tissues during aging^41–43^. As we observed that Avn C showed cytoprotective effect against H_2_O_2_-induced and TNF-α-mediated increase in pro-inflammatory cytokine transcripts, we further investigated if Avn C could also exert this activity under normal metabolic conditions inside cells. Following treatment of 2DD fibroblasts with Avn C for 48 h, RNA was extracted, converted to cDNA and evaluated by qRT-PCR (Fig. 5A). Results demonstrated a significant reduction of *Il-1β, Il-8, IL-6* and *TNF-α* transcripts in the basal level, except for *TNF-α* at 100 μM Avn C treatment condition. Western blot analyses and subsequent densitometry of band intensity revealed that 100 μM and 200 μM Avn C treatment subtly, but significantly decrease of phospho-NF-*κ*B p65 (Ser536) in 2DD fibroblasts (Fig. 5B). To further confirm that Avn C reduced inflammatory response by inhibiting NF-κB activation, chromatin immunoprecipitation-qPCR (ChIP- qPCR) was performed to analyze changes of NF-κB binding activity to promoter regions of *Il-1β* and *Il-8* with 48 hours of 100 μM Avn C treatment. We observed that 100 μM Avn C treatment significantly decreased NF-κB binding activity to both *Il-1β* and *Il-8* promoter regions (Fig. 5C). These observations indicate that the exposure of primary dermal fibroblasts to Avn C leads to decreased NF-κB activation, thus preventing NF-κB binding activity at specific pro-inflammatory cytokine promotes.

### **Avn C induces*heme oxygense-1 (HO-1)*expression through increased DNA binding activity of Nrf2 in normal human skin fibroblasts**

**Figure 6:**
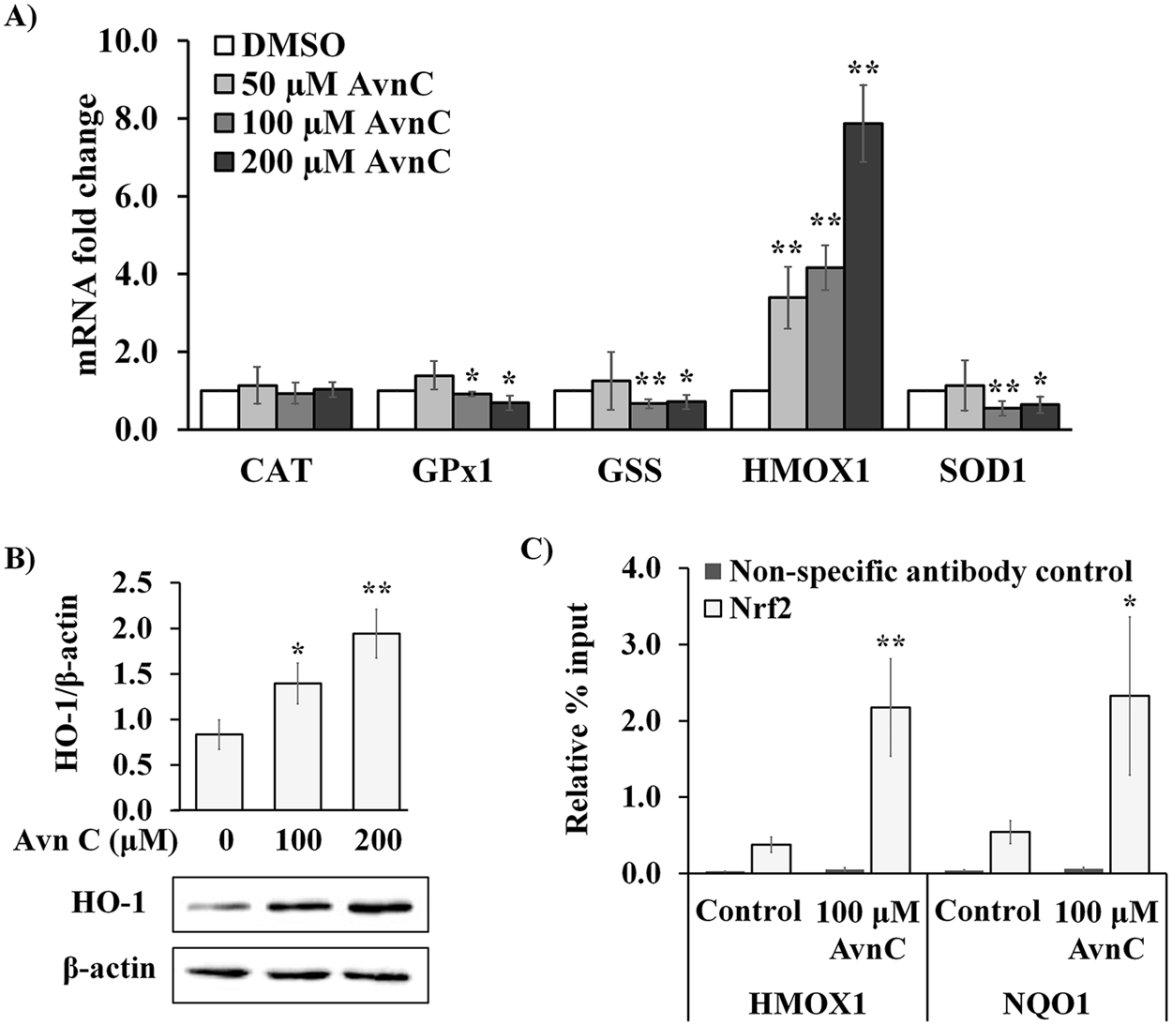
Avn C induces heme oxygense-1 expression through increased DNA binding activity of Nrf2 in 2DD fibroblasts. (**A**) 2DD human skin fibroblasts were treated with DMSO (control) or Avn C (50 μM, 100 μM and 200 μM) for 48 h. qRT-PCR on cDNA libraries was performed for *glutathione synthetase* (*GSS*), *heme oxygenase 1* (*HMOX1*), *superoxide dismutase 1* (*SOD1*), *glutathione superoxidase 1* (*GPx1*) and *catalase* (*CAT*) gene transcripts. Data are represented as fold changes against control. (**B**) Western blot analysis of heme oxygenese-1 (HO-1) expression in 2DD cells treated with DMSO (control) or Avn C (100 μM and 200 μM) for 48 h. Data are presented as a ratio of HO-1 to β-actin. Representative blot images are shown. (**C**) ChIP-qPCR analysis of Nrf2 binding activity to promoter regions of *heme oxygenase 1* (*HMOX1*) and *NAD(P)H quinone dehydrogenase 1 (NQO1)* in 2DD cells treated with DMSO (control) or 100 μM Avn C for 48 h. Data are represented as the enrichment relative to % input. All graphs indicate the average values from three biological replicates. Error bars represent the standard deviations. *p values < 0.05, **p values < 0.01 vs control.

Previous data demonstrates the structure-function relationship of Avn C and Nrf-2 DNA binding in papilloma virus immortalized HK-2 kidney cells^26^. Given that Avn C showed protective effect against H_2_O_2_-induced oxidative stress through free radical scavenging which decreased H_2_O_2_-induced antioxidant gene transcription, we further investigated if Avn C could also exert protective effects by activating other protective pathways in untransformed fibroblasts. 2DD cells were treated with Avn C for 48 h and RNA was extracted, converted to cDNA and evaluated by qRT-PCR to examine if there was any antioxidant enzyme that was up-regulated by Avn C treatment. Transcript quantification by qRT-PCR demonstrated that *HMOX1* was significantly up-regulated by 50, 100 and 200 μM Avn C treatment (Fig. 6A), whereas other antioxidant genes either showed no significant change or significant decreases. To further confirm if *HMOX1* gene expression was induced by Avn C treatment, western blot was used to analyze protein expression levels of HO-1, which is coded by *HMOX1* gene. It was found that 100 and 200 μM Avn C treatment significantly increased protein expression of HO-1 in 2DD fibroblasts (Fig. 6B).

Expression of *HO-1* is regulated by nuclear factor erythroid related factor 2 (Nrf2)^26,44^. To investigate the impact of Avn C treatment on Nrf2 DNA binding activity of, 2DD cells were treated with vehicle (DMSO) or 100 μM Avn C for 48 h. Cells were then cross-linked and chromatin immunoprecipitation-qPCR (ChIP- qPCR) was performed to analyze Nrf2 binding activity to promoter regions of *HMOX1* and *NAD(P)H quinone dehydrogenase 1 (NQO1)* genes, which have been identified as Nrf2-regulated genes^45,46^. Treatment of cells with 100 μM Avn C treatment significantly increased Nrf2 DNA binding activity to both *HMOX1* and *NQO1* promoter regions (Fig. 6C). Taken together, these findings indicate that Avn C induced *HO-1* expression through increased DNA binding activity of Nrf2 in 2DD fibroblasts. This indicates that Avn C not only reduces oxidative stress through scavenging free radicals, it could also function as a mediator that affects signaling pathways for exerting cytoprotective effect in primary dermal fibroblasts.

### Avn C reduces proliferative rate of 2DD fibroblasts through autophagy-independent pathway

Our results indicate that Avn C had cytoprotective effect against oxidative and inflammatory stress through inhibition of NF-κB activity and activation of Nrf2. As many studies have demonstrated that some phenolic compounds slow proliferative rates by activating autophagy pathway, which promoted cellular health and increased longevity^47–49^, we evaluated if Avn C can promote cellular health through the similar mechanisms. To determine the impact of Avn C on 2DD fibroblasts proliferative rates, we treated 2DD cells with either vehicle (DMSO) or 50, 100 and 200 μM Avn C for 48 hours and monitored their growth. Avn C had a significant impact on proliferative rates in 2DD fibroblasts at 50, 100 and 200 μM concentrations (Supplemental Fig. 1A). Trypan blue staining of cells indicated that there was no significant death of any of the treatment at the concentrations outlined (Supplemental Fig. 1B). To further confirm this response, we immuno-labeled Avn C treated 2DD fibroblasts for the proliferative marker Ki-67, which is a nucleolar and chromatin-associated protein only present in actively dividing cells. 2DD cells showed a significant decreased in the number of Ki-67 positive cells at 50, 100 and 200 μM Avn C treatment (Supplemental Fig. 1C). These observations indicate that Avn C is modulating cells growth in primary fibroblasts without causing cells death.

As we observed a significantly decrease of proliferative rate with Avn C treated normal human fibroblast cells, we investigated if this effect was caused by activation of autophagy pathway. Western blot analyses were performed and we observed no change in the levels of the autophagy marker protein light chain 3 isoform II (LC3-II) (Supplemental Fig. 1D). Sirtuin 1 (SIRT1) and mTOR are upstream molecules of autophagy activation and responsible for sensing cellular energy levels. Western blot results also demonstrated that there was no change in SIRT1 and phospho-mTOR (activated mTOR) protein levels following 48 h Avn C treatment (Supplemental Fig. 1D), indicating Avn C reduces proliferative rate of normal human skin fibroblasts through autophagy-independent pathway.

## Discussion

Here we demonstrated that Avn C significantly protected human primary dermal fibroblasts from H_2_O_2_-induced cellular damage shown as reduced levels of intracellular free radicals, DNA damage and pro-inflammatory response. Pre-treatment of Avn C also protected cells against inflammatory responses induced by TNF-α. Furthermore, mechanistic studies revealed that Avn C suppressed basal level of inflammation through decreased NF-κB DNA binding activity and induced *HO-1* expression through increased Nrf2 DNA binding activity, indicating that Avn C exerts antioxidant and anti-inflammatory effect through modulating NF-κB and OH-1/Nrf2 signaling pathways in addition to free radical scavenging.

Recently, Avn C has been demonstrated to attenuate inflammatory pathways in C2C12 muscle cells in response to H_2_O_2_ and TNF-α. Using 30μM Avn C, Yeo and colleagues demonstrate parallel decreases in NF-κb DNA binding activity and decreased TNF-α mediated increase in production of ROS^50^. The use of this concentration, the concentrations we used, as well as that in several other studies (ranging up to 400 μM) would not be considered relevant for physiological applications. Regardless, these concentrations are important for identifying the underlying molecular mechanisms by which Avn C has the potential to function. We justify the use of 100 μM and 200 μM as they gave statistically significant reduction in cell proliferation without causing cell death. We also failed to see a statistically significant decrease in NF-κB phosphorylation at 50 μM (Fig. 5B), further justifying our choice in concentrations. We stress that our observation implicate pathways that are activated/repressed in response to 100 μM and 200 μM and that these concentrations were used as a tool to assess cellular response. These data indicate a potential application of Avn C or the development of structurally similar compounds for the use as potential nutraceuticals or therapeutic agents. Further testing in animal models is required to validate this hypothesis.

Several studies have shown *in vitro* antioxidant activity of Avn C using different model systems, including DPPH (2,2-diphenyl-1-picrylhydrazyl) free radical scavenging activity^4,13^, ferric reducing potential measurement^13^ and inhibition of β-carotene bleaching^13^. Although their results illustrated great antioxidant potential of Avn C, it was still unclear that if this compound can effectively penetrate cells and scavenge intracellular free radicals. In this study, we used normal human skin fibroblast cells to investigate intracellular antioxidant activity of Avn C. Free radicals inside cells were labelled by Mitotracker Orange, which is a non-fluorescent dye in its reduced form. When it interacts with free radicals, it is oxidized and becomes fluorescence. By observing fluorescence intensity, we found that pre-treatment with Avn C reduced free radical levels induced by H_2_O_2_, thus indicating that AVN C is not only able to penetrate cells but to reduce levels of free radical. This is consistent with previous observations indicating that Avn C is able to penetrate cells^51^. We also found Avn C significantly suppressed increased levels of antioxidant enzyme transcripts induced by H_2_O_2_, which confirmed antioxidant effect of Avn C inside cells.

Free radicals and oxidative stress also play major roles in DNA damage and inflammation development. Functional roles of H_2_O_2_ in inflammation have been investigated by several studies. They showed that H_2_O_2_ can acting as signaling molecules that chemically modify protein thiols. As a result, H_2_O_2_ exposure activates inflammatory response through phosphorylation of tyrosine residues in NF-κB and its upstream regulators such as IκB and IκK^38,39^. In addition, cellular damage caused by H_2_O_2_ releases TNF-α from damaged cells, resulting in amplification of pro-inflammatory stimuli^43^. In this study, we observed that H_2_O_2_ induced the transcriptional levels of *Il-1β, Il-8, IL-6* and *TNF-α*, which were significantly reduced by Avn C pre-treatment. These observations indicate that Avn C has the ability to protect 2DD primary dermal cells against free radical-induced inflammatory responses. In addition to inflammation, DNA damage is another major effect of free radicals. Increased levels of DNA damage resulted from increased levels of free radicals leads to increase mutation rates, leading to cancer and variety of age-related disorders^52,53^. Our results demonstrated that pre-treatment with Avn C significantly reduced H_2_O_2_-induced protein expression of γ-H2AX, which is a sensitive marker for DNA double-strand breaks^35^. Our observations further demonstrate that Avn C protects primary dermal cells against free radical induced DNA damage, resulting in an increased DNA stability and function.

Given that pre-treatment of Avn C showed several protective effects against H_2_O_2_-induced cellular injuries, most likely through its free radical scavenging activity, we further investigated if Avn C has impacts on biological pathways independently of exogenous free radical exposure. We used TNF-α instead of H_2_O_2_ to induce pro-inflammatory response. TNF-α can either acting as an autocrine or paracrine stimulator, binds cell receptors and triggers signaling events, leading to NF-*κ*B activation and pro-inflammatory cytokines expression^40^. TNF-α induced the transcriptional levels of *Il-1β, Il-8* and *TNF-α*, which were significantly reduced by Avn C pre-treatment. Notably, only a slight increase in Il-6 transcripts was observed with TNF-α treatment, indicating that exogenous H_2_O_2_ and TNF-α have different impacts on pro-inflammatory markers in primary dermal fibroblasts. Our results suggest that Avn not only acts as radical scavenger inside cells, it can also function as a signaling mediator for anti-inflammatory effect.

Several studies indicate that chronic low-grade inflammation is tightly associated with aging process, including activation of NF-κB system in various tissues^41–43^. It has also been shown that the expression of several inflammatory cytokines was increased during ageing and used as reliable aging parameters, including IL-6, TNF-α and IL-1β^54,55^. As we observed anti-inflammatory effect of Avn C against H_2_O_2_-induced and TNF-α-induced pro-inflammatory responses, we further investigated if Avn C could also reduce basal level of pro-inflammatory activation. Our results demonstrated that Avn C reduced pro-inflammatory cytokine transcripts, phosphorylated NF-κB expression and NF-κB DNA binding activity. Our results are contradictory to a previous study suggesting that Avns do not directly inhibit binding of NF-κB to DNA^23^. However, we used ChIP assay to directly show a decreased binding activity of NF-κB to DNA. Our results demonstrate the repression effect of Avn C on basal low-grade inflammation, although likely not through direct interaction of Avn C with NF-κB.

It has been found that many polyphenols have the ability to up-regulate antioxidant enzymes, which are involved in their antioxidant mechanisms^56–58^. We did not observe up-regulatory effect of Avn C on *SOD1*, *CAT*, *GPx* and *GSS* transcription; however, we found a significant increase of *HO-1* expression. Cumulative evidence has shown that *HO-1* is one of the most critical cytoprotective mechanisms due to its anti-oxidant, anti-inflammatory and anti-apoptotic effects^58^. *HO-1* is a highly inducible form of heme oxygenase, which catabolizes heme and produce biliverdin, ferrous iron (Fe^2+^), and carbon monoxide (CO)^59^. The expression of *HO-1* is regulated by its transcription factor Nrf2. Under non-stress conditions, Nrf2 is sequestered in the cytoplasm by the actin-binding protein, Keap1. Following Keap1 degradation, Nrf2 is translocated from the cytosol to the nucleus to induce gene expression by binding to the antioxidant response element (ARE) and drive the expression of antioxidant enzymes^60,61^. Previous observations by Fu and colleagues^26^ indicate that Avn C induced *HO-1* expression through increased Nrf2 translocation in immortalized kidney epithelial cells. Here, we demonstrated that Avn C up-regulated *HO-1* expression in normal human fibroblasts and further validated increased Nrf2 DNA binding activity by ChIP assay. The decrease in *SOD1*, *CAT*, *GPx* and *GSS* transcripts likely represents the protection of cells by Avn C scavenging radicals and inhibiting NF-κB activation, while stimulating Nrf-2 activation to drive the expression of *HO-1*. The results revealed another possible cytoprotective mechanism of Avn C for its antioxidant and anti-inflammatory effects independent of anti-oxidant enzyme production mediated through NF-κB.

Numerous studies (reviewed in^10^) have used cancer cell lines to demonstrate the anti-proliferative effects of Avns, focusing on the potential use of these compounds as therapeutic agents for treating cancer. In our study, Avn C slowed the growth of normal dermal fibroblasts as well; however, this decrease in proliferation without causing cell death can be beneficial. Treatment of cells with putative anti-aging compounds such as rapamycin and metformin also decrease proliferative rates of normal dermal fibroblasts and is associated with increased cellular health^62,63^. This increase in cellular health is linked to processes such as autophagy and increased protein homeostasis. Although Avn C treatment did not lead to a detectable increase in the autophagy marker LC3-II by western blot, the decrease in proliferative rates are consistent with increased cellular health. Our observations of decreased intracellular free radicals, decreased levels of NF-κB DNA binding and increased Nrf-2 DNA binding do, although indirectly, support this hypothesis.

In conclusion, we found that Avn C protected normal primary cells from oxidative stress, DNA damage and inflammatory response. Furthermore, we demonstrated that those cytoprotective effects were associated with its free radical scavenging activity, pro-inflammatory cytokine suppression, NF-κB inhibition, *HO-1* induction and Nrf2 activation in normal dermal fibroblasts and that Nrf-2 is directly binding to chromatin in response to Avn C. Taken together, Avn C exhibits multidirectional cytoprotective capabilities that contribute to an overall improvement of cellular health. Further investigation in animal models using physiologically relevant concentrations of this compound will validate our conclusions and further support the use of Avn C as a potential nutraceutical.

## Materials and Methods

### Cell culture

Normal human skin fibroblasts 2DD cells (previously described in Bridger *et al*. 1993) were grown in high glucose (4.5 mg/mL) Dulbecco’s Modified Eagle Medium (DMEM) (pH 7.7; Corning, USA, Cat #: ca45000-304) supplemented with 10% fetal bovine serum (FBS) (Gibco, Thermo Fisher Scientific, USA, Cat #: 12483-020) and 1.0% penicillin streptomycin (GE Healthcare Life Sciences, USA, Cat #: SV30010). Cells were seeded at an initial density of 3000 cells/cm^2^ and incubated at 37 °C in a humidified atmosphere with 5% carbon dioxide (CO_2_). Cells were passaged every 3 to 4 days with cells never excessing 80% confluency. Cells from passage number 12 to18 were used in experiments.

### Cell treatment

Media used for treatments was identical to that used for cell culture.

#### Cell treatment with Avn C

Avn C was dissolved in dimethyl sulfoxide (DMSO). The final concentrations of DMSO in culture media were 0.4%. 2DD cells were seeded at a density of 3000 cells/cm^2^. After 24 hours, DMSO or Avn C (50, 100 and 200 μM) were added into culture media and incubate at 37 °C & 5% CO_2_ for 48 h. Several concentrations of Avn C were tested on 2DD cells. Concentrations of 100 μM and 200 μM showed the most dramatic decrease on cell proliferation rates without causing cell death. Furthermore, several previously published articles use concentrations of Avn C within this range as tools to demonstrate mechanisms. Although not physiologically achievable, these concentrations can be used as tools to identify the mechanisms by which Avn C maybe useful for manipulating biological systems.

#### *H*_2_*O*_2_*-induced cellular stress*

2DD cells were seeded at a density of 3000 cells/cm^2^. After 24 hours, DMSO or Avn C (100 and 200 μM) were added into culture media and incubated at 37 °C & 5% CO_2_ for 48 h. Cells were then washed two times with serum-free DMEM and H_2_O_2_ (Sigma-Aldrich, USA, Cat #: 216763) was added to serum-free DMEM media at a final concentration of 200 μM and incubate at 37 °C & 5% CO_2_ for 1 h.

#### TNFα-induced cellular stress

2DD cells were seeded at a density of 3000 cells/cm^2^. Cells were treated with DMSO or Avn C (100 and 200 μM) for 24 hours. TNFα was then added to media to a final concentration of 10 ng/mL and incubate for 4 hours to induce inflammatory response.

### Cell counts and cell viability

2DD cells were grown in 6-well plates under the previously described treatment conditions. At the end of incubation period, cells were dissociated from the surface of culture plates using TrypLE Express (Life Technologies, USA, Cat #: 12604013) and centrifuged at 200 × g for 5 min to pellet cells. Cells were then re-suspended in culture media. Cell counts were conducted using 10 µL of cell suspension on a 0.0025 mm^2^ Neubauer improved haemocytometer. For cell viability test, cell suspensions were mixed 1:1 with 0.4% (v:v) trypan blue dye (VWR international, USA, Cat #: CA97063-702). Total stained and unstained cells were counted by a haemocytometer under light microscope. Cells stained dark blue were considered not viable.

### Immuno-labeling of Ki67

2DD cells were grown on sterilized 22 mm^2^ glass coverslips under the previously described conditions. At the end of incubation period, cells were fixed with 3.7% formaldehyde in PBS for 10 min at room temperature followed by incubation in ice-old methanol/acetone (1:1 mixture) for 8 min at 4 °C. Cells were washed two times with 1 X PBS and then permeabilized with 0.5% Triton X-100/PBS for 10 min at room temperature. After that, cells were blocked with 1% BSA for 1 h at room temperature. Following this, cells on coverslips were incubated in rabbit anti-Ki67 (1:2000 dilution; Novacastra, USA, Cat #: NCL-Ki67) diluted in 1.0% BSA/PBS for 1 h in a dark humidity chamber. Cells were then washed two times with PBS and incubated in goat anti-rabbit A488 (1:200 dilution; Stratech/Jackson Scientific, UK, Cat #: 111545-003-JIR) diluted in 1.0% BSA/PBS for 1 h in a dark humidity chamber. Nuclei were counterstained with VECTASHIELD Mounting Medium with DAPI (Vector Laboratories, USA, Cat #: H1200), mounted onto glass slides and sealed with nail varnish. Images were collected at 40X magnification with a constant exposure time. Image analyses were conducted by Image J software and a threshold was selected for all images. Any nuclei containing label above the threshold was scored as positive for Ki67.

### Mitotracker Orange labeling of intracellular free radicals

MitoTracker Orange CM-H_2_TMRos (Molecular Probe, Eugene, Cat #: M-7511) is a reduced, non-fluorescent dye that stains mitochondria in live cells and becomes fluorescent when oxidized. 2DD cells were grown on sterilized 22 mm^2^ glass coverslips under the previously described conditions. At the end of incubation period, cells were stained with MitoTracker Orange according to the manufacturer’s instructions. Briefly, coverslips with adherent cells were washed two times with serum-free media. MitoTracker Orange was dissolved in DMSO and then added to the serum-free media at a final concentration of 500 nM. After incubating for 30 min, cells on coverslips were fixed with 3.7% formaldehyde in phosphate buffered saline (PBS) for 10 min and permeabilized with 0.5% Triton X-100/PBS for 10 min at room temperature. Nuclei were counterstained with VECTASHIELD Mounting Medium with DAPI (Vector Laboratories, USA, Cat #: H1200), mounted onto glass slides and sealed with nail varnish. Images were collected at 40X magnification with a constant exposure time. Image analyses were conducted by Image J software.

### RNA extraction, cDNA library synthesis and RT-qPCR

2DD cells were grown in 10 cm diameter plates under the previously described treatment conditions. RNA was extracted using TRIzol reagent (Life Technologies, USA, Cat #: 15596026) according to manufacturer’s instructions. RNA concentration was determined using NanoDrop™ 2000 Spectrophotometers (Thermo Fisher Scientific, Wilmington, USA). The quality and integrity of RNA samples was determined by running RNA samples on a 1% agarose gel. RNA samples that showed sharp and clear 28 S and 18 S ribosomal RNA (rRNA) bands were used for cDNA synthesis. Two micrograms of RNA from each treatment were used in cDNA synthesis reactions in combination with random hexamers (50 ng per reaction) and Superscript III (Invitrogen) as per manufacturer’s instructions. The final reaction was diluted to a final volume of 200 μl.

RT-qPCR was conducted using RotorGene qPCR machine (Qiagen). The sequences of the primers used for qPCR were designed by using Primer 3 (version 4.0) software and summarized in Table 1. Ten microliter reactions were set up using 5 μl SYBR Green SuperMix 2x (Quantabio), 2 μl cDNA template, 2 μl H_2_O and 1 μl of 3 μM forward and reverse primers. All reactions for each gene were run in triplicate with non-template controls. Results were quantified using the ΔΔCt method against two normalizing genes: *SPARC* and *FKBP10*. The fold change values were calculated from ΔΔCt method for antioxidant genes expressed in treatment vs control condition.

**Table 1:** Primers for RT-qPCR.

Gene	Forward primer (5′ → 3′)	Reverse primer (5′ → 3′)	Amplicon (bp)
**Normalizing genes**			
SPARC	TACATCGGGCCTTGCAAATAC	GGTGACCAGGACGTTCTTGAG	99
FKBP10	GCCGTGCTAATCTTCAACGTC	GGTGGTCTCATTGCAGGTCTC	105
**Antioxidant enzyme genes**			
GSS	AGCTTTCCATCTGAGGACCAG	TCCTATCCCAAGTCAGGCACT	188
HMOX1	AAAGGAGGAAGGAGCCTATGG	AGCTGCCACATTAGGGTGTCT	149
SOD1	GGCAAAGGTGGAAATGAAGAA	GGGCCTCAGACTACATCCAAG	112
GPx1	CCTCCCCTTACAGTGCTTGTT	GAGAAGGCATACACCGACTGG	115
CAT	TGCAAGCTAGTGGCTTCAAAA	TCCAATCATCCGTCAAAACAA	143
**Pro-inflammatory cytokine genes**			
IL-1β	GCTACGAATCTCCGACCACCA	AACCAGCATCTTCCTCAGCTTG	91
IL-6	CGTCCGTAGTTTCCTTCTAGCTT	CAAAGGAGGACCTTGTGGCA	103
IL-8	TGCAGTTTTGCCAAGGAGTG	TGATAAATTTGGGGTGGAAAGG	83
TNFα	CAATGGCGTGGAGCTGAGAG	TCTGGTAGGAGACGGCGATG	152

### Protein extraction and Western blot analysis

Western blot analyses were performed on cell lysates obtained from the previously described treatment conditions. Whole cell lysates were extracted using Laemmli lysis buffer (62.5 mM Tris-HCl pH 6.8, 2% SDS, 10% glycerol and 5% 2-mercaptoethanol) containing Protease Inhibitor Cocktail 2 (Sigma-Aldrich, USA, Cat #: P8340) and Phosphatase Inhibitor Cocktail 2 (Sigma-Aldrich, USA, Cat #: P5726) at a ratio of 100:1:1. Equivalent denatured cell lysates (15–30 µg cell lysate per sample) were loaded and separated by SDS‐PAGE and transferred to nitrocellulose membrane (Bio-Rad Laboratories, CA, Cat #: 1620115). The membranes were then blocked in 5.0% skim milk powder in phosphate buffered saline containing 0.05% Tween 20 (PBST), followed by incubating overnight at 4 °C in primary antibody diluted in 5.0% skim milk powder in PBST. Primary antibodies used were rabbit anti-phospho-NF-κB p65 (Ser536) antibody (1: 1000 dilution; Cell Signaling Technology, USA, Cat #: 3033), mouse anti-SIRT1 antibody (1:1000 dilution; Abcam, CA, Cat #: ab110304), mouse anti-phospho-mTOR (Ser 2448) antibody (1: 500 dilution; Santa Cruz, USA, Cat #: sc-293133), rabbit anti-LC3II (1:1000 dilution; Abcam, CA, Cat #: ab48394), mouse anti-heme oxygenase 1 antibody (1: 500 dilution; Santa Cruz, USA, Cat #: sc-136960), mouse anti p-Histone H2A.X (Ser 139) antibody (1: 500 dilution; Santa Cruz, USA, Cat #: sc-517348) and rabbit anti-β-actin (1:2000 dilution; Abcam, CA, Cat #: ab8227). Following primary antibody incubation, membranes were washed and incubated in secondary antibody diluted in 5.0% skim milk in PBST. Secondary antibodies used were goat anti-rabbit horse radish peroxidase (HRP) (1:2000 dilution; Abcam, CA, Cat #: ab97069) or donkey anti-mouse HRP (1:2000 dilution, Jackson Scientific, UK, Cat #: 715-035-150). Protein bands were visualized with chemiluminescence using ECL reagent (100 mM Tris-HCl pH 8.5, 0.2 mM p-coumaric acid, 1.25 mM 3-aminophtalhydrazide and 0.1% H_2_O_2_). β-actin was used as a loading control.

### Chromatin immunoprecipitation-qPCR (ChIP-qPCR)

2DD cells were grown in 15 cm diameter plates and treated with DMSO as control or 100 µM Avn C for 48 hours. At the end of treatment period, cells were fixed with 1% paraformaldehyde (Electron Microscopy Sciences, USA, Cat #: 15714) for 10 min at room temperature. Cells were then scraped in ice-cold PBS. Cell pellets were resuspended in ChIP lysis buffer (1% SDS, 10 mM EDTA, 50 mM Tris-HCl pH 8.0) containing Protease Inhibitor Cocktail 2 (Sigma-Aldrich, USA, Cat #: P8340) and Phosphatase Inhibitor Cocktail 2 (Sigma-Aldrich, USA, Cat #: P5726). Following 10 min of incubation on ice, cells were sonicated into DNA fragments with 200–1000 bp in length. 2.5 µg of mouse anti-Nrf2 antibody (Santa Cruz, USA, Cat #: sc-365949) or 2.5 µg of rabbit anti-phospho-NF-κB p65 (Ser536) antibody (Cell Signaling Technology, USA, Cat #: 3033) was added to sheared chromatin samples diluted 10 times in ChIP buffer (0.01% SDS, 1.1% Triton X100, 1.2 mM EDTA, 16.7 mM Tris-HCl pH 8.0 and 167 mM NaCl) containing Protease Inhibitor Cocktail 2 (Sigma-Aldrich, USA, Cat #: P8340) and Phosphatase Inhibitor Cocktail 2 (Sigma-Aldrich, USA, Cat #: P5726). 2.5 µg donkey anti-mouse HRP (Jackson Scientific, UK, Cat #: 715-035-150) was used as non-specific antibody control. The mixture was incubated at 4 °C overnight with rotation, followed by binding to 50 µL Dynabead Protein A (Invitrogen, USA, Cat #: 10006D) at 4 °C for 1 hour. The samples were then washed with ChIP washing buffer I (0.1% SDS, 1% Triton X100, 2 mM EDTA, 20 mM Tris pH 8.0 and 150 mM NaCl), ChIP washing buffer II (0.1% SDS, 1% Triton X100, 2 mM EDTA, 20 mM Tris pH 8.0 and 500 mM NaCl) and ChIP washing buffer III (1 mM EDTA and 10 mM Tris-HCl pH 8.0). After washing steps, samples were eluted with 500 µL freshly made elution buffer (1% SDS and 0.1 M NaHCO_3_) for 1 hour at room temperature. Crosslinks were reversed by adding 200 mM NaCl, 12.5 mM EDTA and 2 µl proteinase K (Invitrogen, USA, Cat #: 25530049), followed by incubating at 65 °C for 5 hours agitating at 900 rpm. DNA from each sample was extracted by phenol-chloroform extraction. qPCR was performed following DNA extraction. Ten microliter reactions were set up using 5 μL PerfeCTa^®^ SYBR^®^ Green SuperMix for iQ (Quantabio, USA, Cat #: 95053-500), 1 μL ChIP DNA sample, 3 μL H_2_O and 1 μL of 3 μM forward and reverse ChIP primers. qPCR reactions were conducted using the Rotor-Gene Q qPCR machine (QIAGEN). All reactions for each gene were run in triplicate with non-template controls. ChIP-qPCR data was normalized by the percent input method. Standard error was calculated as a function of the standard deviations between triplicates. ChIP-qPCR primers used are listed in Table 2.

**Table 2:** ChIP-qPCR primers.

Gene	Forward primer (5′ → 3′)	Reverse primer (5′ → 3′)	Amplicon (bp)
HMOX1	CCGCCCCGAGATCTGTTTTC	ATGTCCCGACTCCAGACTCC	157
NQO1	TGAGAGTCCTGGGGAGACAT	CACCCAGGGAAGTGTGTTGT	103
IL-1β	TTGCCCTTCCATGAACCAGAG	AAGCAGAAGTAGGAGGCTGAGA	147
IL-8	GTGATGACTCAGGTTTGCCCT	CTTATGGAGTGCTCCGGTGG	139

### Statistical analysis

Results were presented as mean ± standard deviation of three biological replicates. Two-tailed Student’s *t*-test was performed to assess statistical significance between groups as indicated in the legends. *p*‐values < 0.05 were considered statistically significant.

## Supplementary information

Avn C reduces proliferative rate of 2DD fibroblasts through autophagy-independent pathway.
