# Supplementary material for: Cytoprotective effects of Avenathramide C against oxidative and inflammatory stress in normal human dermal fibroblasts
Source: Sci Rep. 2019 Feb 27;9:2932. doi: 10.1038/s41598-019-39244-9 (PMC6393498; doi:10.1038/s41598-019-39244-9)

**Title:** Cytoprotective effects of Avenathramide C against oxidative and inflammatory stress in normal human dermal fibroblasts.

Chenxuan Wang^1^ and Christopher H. Eskiw^1,2^

^1^ Department of Food and Bioproduct Sciences, University of Saskatchewan

^2^ Department of Biochemistry, University of Saskatchewan

Corresponding Author: Christopher Eskiw ([c.eskiw@usask.ca](mailto:c.eskiw@usask.ca))


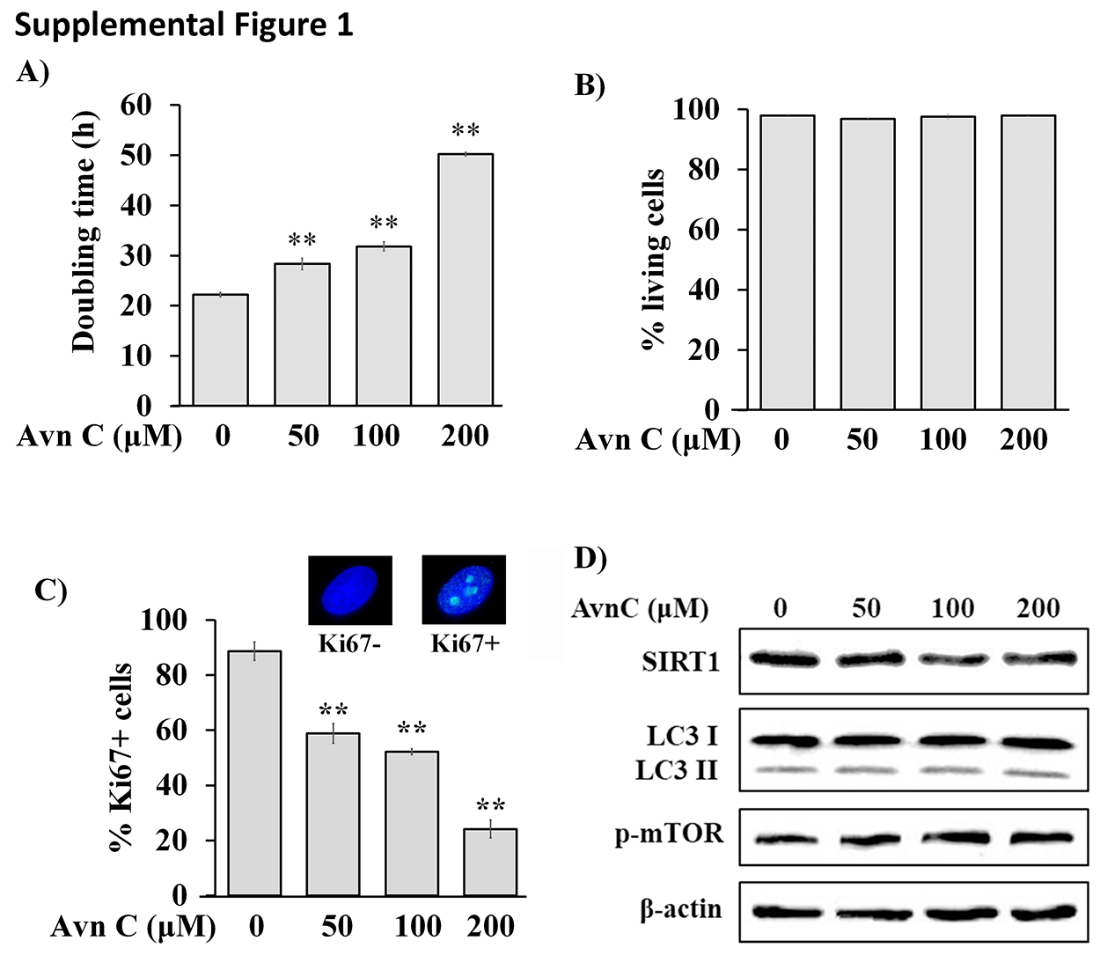

Supplement: Supplementary file 1 — Avn C reduces proliferative rate of 2DD fibroblasts through autophagy-independent pathway. [file 41598_2019_39244_MOESM1_ESM.docx]
